# Sexual Self-Esteem and Psychological Burden of Adults With Neurofibromatosis Type 1

**DOI:** 10.3389/fpsyg.2022.883019

**Published:** 2022-06-09

**Authors:** Anna Leidger, Marie Vosschulte, Timo O. Nieder, Victor-Felix Mautner

**Affiliations:** ^1^Clinic and Polyclinic of Neurology, Neurofibromatosis Outpatient Department, University Medical Center Hamburg-Eppendorf, Hamburg, Germany; ^2^Institute for Sex Research, Sexual Medicine and Forensic Psychiatry, University Medical Center Hamburg-Eppendorf, Hamburg, Germany

**Keywords:** chronic illness, neurofibromatosis type, sexuality, quality of life, self-esteem

## Abstract

Neurofibromatosis type 1 (NF1) is one of the most common tumor predisposition syndromes which primarily affects the skin. NF1 is characterized by various degrees of skin tumors and pigmentation abnormalities such as café-au-lait macules. Other skin diseases, such as psoriasis or neurodermatitis, have a negative influence on sexuality and quality of life and represent a psychological burden for those affected. The present study investigated the extent to which skin tumors (disfigurement) are related to sexuality and psychosocial factors in NF1 individuals. An anonymous online survey was carried out on Facebook and the webpage of the German Neurofibromatosis Association and a total of 166 persons participated. Of these participants, 92 were affected by NF1.74 healthy persons took part in the survey as a comparative group. Results show a significant relation between sexuality, body image, quality of life and the presence of depressive symptoms of the NF1 affected persons. Individuals with NF1 show a more negative sexual self-esteem. These concerns should be taken into account in NF1- related health care approaches.

## Introduction

Skin diseases such as psoriasis, atopic dermatitis, and acne inversa significantly influence the body image, sexuality and self-perception of those affected (Niemeier et al., 1997; Seikowski et al., 2008; Magin et al., 2010; Köhn et al., 2015; Ryan et al., 2015). Neurofibromatosis Type 1 (NF1) as a multisystem genetic disorder affecting the skin in almost all individuals concerned (Zimpfer et al., 2004). NF1 is an autosomal dominant genetic disorder with a prevalence of 1: 2500–3000 (Lammert et al., 2005). The visible characteristics are hyperpigmentation’s of the skin (multiple café-au-lait spots, axillary and inguinal freckling) and disfiguring tumors (neurofibromas -benign cutaneous and/or subcutaneous tumors) (Kehrer-Sawatzki and Mautner, 2009). Neurofibromas tend to grow slowly but chronically with increasing age, especially during adolescence.

It is known that people affected by NF1 tend to have a higher psychological burden and report about more affective symptoms, for example anxiety or depression (Reichardt et al., 2013). It is also known that the visibility of cutaneous neurofibromas are also associated with major depression and a greater stress experience (Cohen et al., 2015). Research has found that the effects of disease visibility on psychological burden are mediated by the body image of the NF1 patients (Granström et al., 2012). Furthermore, NF1 individuals demonstrate a decreased quality of life compared to healthy controls (Kodra et al., 2009; Vranceanu et al., 2013). Current findings suggest experimentally, the higher the use of dysfunctional coping, the more quality of life, psychological stress and self- esteem are affected (Bottesi et al., 2020).

A cohort cross-sectional study carried out by the NF Outpatient Clinic at the University Medical Center Hamburg-Eppendorf, collected questionnaire information about sexuality of 45 NF1-affected people (Leidger et al., 2019). 13 persons (29%) reported to be impaired by visible and palpable neurofibromas in the genital area or on the genitals. Female patients felt more affected than male patients. The sexual functions were unremarkable. Furthermore, the clinical or neurological severity of neurofibromatosis seemed not to be associated with sexual satisfaction. A tendency of a relation between being in an intimate partnership and sexual satisfaction has been identified (Leidger et al., 2019). An intimate partnership was considered as a productive factor in managing sexuality- related issues in persons affected by NF1. Leidger et al. (2019) considered counseling on sexuality and support in finding a partner to be probably useful within NF1 treatment. Overall, no physiological impairment of the sexuality was found. Nevertheless, people affected by NF1 tend to feel impaired in their sexuality and their sexual perception (Leidger et al., 2019) while these issues are important aspects of quality of life and have furthermore an impact on the mental health. However, so far, intimacy and sexuality of individuals affected by NF1 has been insufficiently investigated.

Therefore, the present study was developed to identify the impact of NF1 on the sexual self- esteem and possible psychological burden. Sexual self-esteem is defined as the self-assurance going along with sexual activities and the perception of its own sex appeal. Psychological burden means the burden and the insecurity which comes along with visible neurofibromas and the disease in general (Reichardt et al., 2013; Leidger et al., 2019) and is defined as affective symptoms. The impact of the disease, disease visibility, sexuality and subjective attractiveness, sexuality in general, affective symptoms and the quality of life were defined as the variables to be captured. We first hypothesized that people with NF1 are more affected in their sexuality and sexual self-esteem, the affective symptomatic and the quality of life compared to a healthy control-group. Secondly we hypothesized that the disease NF1 and the perception of the visibility of the symptoms as far as the subjective attractiveness have an impact on the sexuality and sexual self-esteem, the affective symptomatic and the quality of life: The more the disease and perceived visibility and the less the subjective attractiveness are pronounced the more the sexual self-esteem and perception is impaired, the more affective symptoms are reported and the lower the quality of life. We measured data with different standardized questionnaires and one self-developed questionnaire.

Healthcare providers hardly address intimacy concerns in the NF1 community (Cannon et al., 2019). Nevertheless, this topic might be relevant due to the fact that sexuality is an important aspect of the quality of life and might impact other issues of the mental health.

## What’s Already Known About This Topic?

It is known that psychological burden is higher in NF1 affected people.

Major depression and greater stress experience are also associated with the visibility of cutaneous neurofibromas. Research has found that the effects of disease visibility on psychological burden are mediated by the body image of the NF1 patients. NF1 individuals demonstrate a decreased quality of life when compared to healthy controls and reported to be impaired by visible and palpable neurofibromas in the genital area or on the genitals.

## What Does This Study Add?

Neurofibromatosis type 1 affected people have a lower sexual self-esteem and a more negative body image compared to a comparative group.

There is clearly a need of sexuality-related counseling and interventions if indicated.

## Summary

### Background

Neurofibromatosis type 1 (NF1) is one of the most common tumor predisposition syndromes which primarily affects the skin. NF1 is characterized by various degrees of skin tumors and pigmentation abnormalities such as café-au-lait macules. Other skin diseases, such as psoriasis or neurodermatitis, have a negative influence on sexuality and quality of life and represent a psychological burden for those affected.

### Objectives

To investigate whether sexual self-esteem, quality of Life and psychological burden are influenced by the disease in a sample of individuals with neurofibromatosis type 1 compared to a healthy group.

### Methods

Ninety-two adults with NF1 and seventy-four healthy persons completed the following questionnaires: The German version of the Beck’s Depression Inventory II (BDI-II); the World Health Organization Quality of Life -BREF (WHOQOL-BREF); the German version of The Multidimensional Sexuality Questionnaire (MSQ) and a self-developed questionnaire concerning neurofibromatosis and sexuality.

Differences in the examined factors between NF1 patients and the comparative group were analyzed with a *t*-test for independent samples and a Mann–Whitney-*U*-test.

Secondly, a multiple linear regression was made to identify the influence of the perceived visibility and subjective attractiveness of NF1 patients on sexual self- esteem, body image, quality of life and affective symptoms.

### Results

A statistically relevant relation between the visible symptoms of the disease and sexuality could been shown. NF1 affected people have a lower sexual self-esteem and a more negative body image compared to a comparative group.

Furthermore, there was a moderate effect of disease visibility and subjective attractiveness on sexual self-esteem, body image, affective symptoms and quality of life.

### Conclusion

The results of the present study suggest that sexuality in NF1 patients has been insufficiently understood so far. There is clearly a need of sexuality-related counseling and interventions if indicated.

## Materials and Methods

This study was an online-based cross-sectional study which has been implemented in the period from May 2019 until July 2019 *via* LimeSurvery.com. The study link was shared on Facebook and the webpage of the German Neurofibromatosis Association (Bundesverband Neurofibromatose)^1^ and referred to in the member’s magazine (Edition June 2019). This study was approved by the ethics commission of the Hamburg medical association. Data was collected online with a set of both the following standardized and self- developed questionnaires. The sexuality in general was measured with the German version of the Multidimensional Sexuality Questionnaire (MSQ; Brenk-Franz and Strauß, 2011); the German version of the Beck’s Depression Inventory II (BDI-II; Beck et al., 1996) was applied to measure the affective symptoms and the World Health Organization Quality of Life -BREF (WHOQOL-BREF; Angermeyer et al., 2000) was used to measure the quality of life. The self-developed questionnaire was designed in collaboration between psychologists and a professor from the Neurofibromatosis Outpatient Clinic and used for the measurement of the disease NF1 and disease visibility and sexuality. It was based on the questionnaire of the pilot study (Leidger et al., 2019) and was validated from 15 participants in the neurofibromatosis outpatient clinic. The cohort of participants and the questionnaires are described below.

### Participants

All participants with NF1 and the healthy controls were between 25 and 55 years old. There was a total of 166 participants. 77 participated in the control group and 92 participated in the NF1 group. Including criteria were the age between 25 and 55 years and for the group with NF1 the existence of the diagnosis NF1. Excluding criteria were the diagnosis of NF2 or Schwannomatosis and younger or older age then defined.

### Measures Demographic Data

The demographic questionnaire collected social characteristics, such as gender, age, current education, number of children, and current employment.

### Disease Visibility, Sexuality and Subjective Attractiveness

This questionnaire contains 13 open questions and 10 statements that can be answered with a five-point Likert Scale. The possible answers are the following: 1: does not apply at all, 2: hardly applies, 3: is applicable to something, 4: applies moderately, and 5: applies completely. The open questions relate to the topics of visibility of neurofibromas (“Do the neurofibromas make you feel uncomfortable?”), sexual activity (“How many sexual partners do you have?”), sexuality within the family (“Was sexuality openly communicated in your family?”), and medical advice on sexuality (“Do you think that a doctor should routinely ask his patients about their sex life?”). The 10 statements refer to the burden of neurofibroma (“I feel ashamed of the visible features of the NF1”), physical and subjective attractiveness (“I feel physically attractive despite my neurofibromas”), sexual relations (“I am afraid of sexual relationships because of my neurofibromas”), sexual functions (“I feel that my sexual function is impaired by the neurofibromas”), and dealing with the disease (“I do not want to have children because of the NF1”). A mandatory answer was not provided, as this could represent a possible inhibition for the intimate subject matter.

### Sexuality

The German version of the Multidimensional Sexuality Questionnaire (MSQ) was used to assess the sexuality of the participants. It’s a measure for self-reporting of psychological tendencies in connection with human sexuality (Brenk-Franz and Strauß, 2011). The questionnaire includes the following scales: Sexual Self-Esteem, Sexual- Preoccupation, Internal-Sexual Control, Sexual-Consciousness, Sexual-Motivation, Sexual-Anxiety, Sexual-Assertiveness, Sexual-Depression, External-Sexual Control, Self-Monitoring, Fear of Sex, Sexual-Satisfaction. Sexual Self-Esteem was the most important scale in the study. It refers to the generalized tendency to positively evaluate the ability to enter into a sexual relationship with another person (Brenk-Franz and Strauß, 2011). The overall reliability of the M-MSQ was found in internal consistency with Cronbach’s α = 0.900–0.931.

### Affective Symptoms

Affective Symptoms were measured with the German version of the Beck’s Depression Inventory II (BDI-II; Beck et al., 1996). The BDI-II aims to measure the severity of depression and consists of 21 questions to be self-reported. It includes items relating symptoms of depression such as hopelessness and irritability, conditions such as guilt or feelings of being punished, as well as physical symptoms like fatigue, weight loss and lack of sexual interest. Each answer scored on a scale value of 0–3, with higher total scores indicating more severe symptoms of depression. The standardized cut-offs are:

0–3: minimal depression.

14–19: mild depression.

20–28: moderate depression.

29–63: severe depression.

Cronbach’s α was found with 0.91.

### Quality of Life

The quality of life was measured with the World Health Organization Quality of Life - BREF (WHOQOL-BREF; Angermeyer et al., 2000). This questionnaire has been developed to provide a short form quality of life assessment, using data from WHOQOL-100 from the WHO. The WHOQOL-BREF contains a total of 26 questions to be self-reported using a five- point Likert-scale. It is composed of four domains. Domain 1 assesses Physical health, which includes daily activities, sleeping, resting, and work capacity. Domain 2: Psychological, includes bodily image and appearance, self-esteem, thinking, learning, memory, and concentration. The third domain, Social relationships, includes personal relationships, social support, and sexual activity. Finally, the fourth domain, Environment, includes financial resources, freedom, physical safety and security. The overall reliability was found in internal consistency with Cronbach’s α = 0.89.

### Statistical Analysis

The statistical analysis was performed using the statistical software IBM SPSS Statistics 25.0. Differences in the examined factors between NF1 patients and the control group were analyzed with a *t*-test for independent samples and a Mann–Whitney-*U*-test. Effect sizes of each significant test were estimated and interpreted by Cohen (1988). Effect sizes of *d* = 0.3 represent weak, *d* = 0.5 medium, and *d* = 0.8 strong effects. Secondly, a multiple linear regression was made to identify the influence of the perceived visibility and subjective attractiveness of NF1 patients on sexual self- esteem, body image, quality of life and affective symptoms. The conditions for multiple regressions were met (Linear relationship, homoscedasticity, independent errors, variance in all predictors, multicollinearity, normally distributed residuals). Six cases in the NF1-Patients and six cases in the comparative group were not included due to the excluding criteria of age.

## Results

A total of *N* = 166 subjects participated in the study. The group of NF1 patients (*n* = 86) consisted of 47 women (54.7%) and 25 men (29.1%) with an average age of 38 years (25–55). Information on gender was missing for 14 participants (16.3%). Fifty NF1- patients (58.1%) were in a stable partnership, 25 participants (29.1%) were single and 11 participants (12.8%) did not provide any information.

The comparative group (*n* = 66) consisted of 45 women (66.2%) and 18 (26.5%) men with an average age of 30 (minimum 25; maximum 52). Information on gender was missing for 5 people (7.4%). 47 participants (69.1%) reported to be in a stable partnership, 17 participants (21%) with 4 people (5.9%) not providing any information on partnership status.

The dropout rate among NF1 patients is 71.78 and 54.60% among the control group.

### Comparison of the Quality of Live, Sexual Self-Esteem, Body-Image and Psychological Burden of Patients With NF1 With the Control Group

Differences in the examined factors between NF1 patients and the comparative group were analyzed with a *t*-test for independent samples and a Mann–Whitney-*U*-test.

There was a significant difference between quality of life of the NF1 group and the control group with a strong effect [*t*(46) = −5.727, *p* < 0.001, *d* = 1.688 95% CI −4.165 to −2.028]. NF1 patients reported a lower quality of life than the healthy control group.

A Mann–Whitney-*U* test was calculated to determine if there were differences in sexual self-esteem, affective symptoms and body image between NF1 patients and the control group. There was a significant difference in sexual self-esteem between both groups, *U* = 1834.5, *Z* = −2.948, *p* = 0.003. The control group has higher values in the variable sexual self-esteem than NF1 patients (Table 1). Lower values correspond to a more negative sexual self-esteem.

**TABLE 1:** Mean value and standard deviation.

	Control group			
NF1	*M*	*SD*	*M*	*SD*
Sexual self-esteem	15.07	2.84	16.31	2.17
Body image	13.05	3.38	15.6	2.33
Affective symptoms	18.23	13.42	7.81	8.20

In addition, there was a significant difference in affective symptoms between both groups, *U* = 1175, *Z* = −5.4, *p* < 0.001. The control group showed lower scores for the variable affective symptoms than NF1 patients (Table 1). Lower values reflect less pronounced depressive symptoms.

Furthermore, there was a significant difference in body image between both groups, *U* = 1438, *Z* = −4.906, *p* < 0.001, with the control group scoring higher in the variable body image than NF1 patients (Table 1).

Neurofibromatosis type 1 patients with a partnership seem to have higher values in the variable sexual self-esteem (mean ± SD 15.39 ± 2.68) than patients without a partnership (mean ± SD 14.71 ± 3.15), but this difference in sexual self-esteem between NF1 individuals being in a partnership and those not was not significant [*t*(70) = 0.967, *p* = 0.337 95% CI −0.730 to 2.106].

### Perceived Disease Visibility and Subjective Attractiveness and Sexuality, Body Image, Affective Symptoms and Quality of Life

A multiple linear regression was made to identify the influence of the perceived visibility and subjective attractiveness of NF1 patients on sexual self-esteem, body image, quality of life and affective symptoms.

10.7% of the variance of sexual self-esteem is explained by the perceived visibility and subjective attractiveness. The model has a medium quality of fit (Cohen, 1988). A positive significant correlation between sexual self-esteem and subjective visibility of characteristics and subjective attractiveness was found.

Furthermore, 40.6% of the variance of body image is explained by the perceived visibility and subjective attractiveness. The model has a strong quality of fit (Cohen, 1988). It has been found that perceived visibility (β = 0.193, *p* = 0.037, 95% CI 0.082–2.519) and subjective attractiveness (β = 0.551, *p* = < 0.001, 95% CI 1.028–2.036) significantly influence the body image.

34.4% of the variance of the affective symptoms is explained by the perceived visibility and subjective attractiveness. The model has a strong quality of fit (Cohen, 1988). It has been found that subjective attractiveness and perceived visibility significantly influences affective symptoms at significance level 0.05 (β = −0.493, *p* = < 0.001, 95% CI − 7.361 to −3.182; β = −0.196, *p* = 0.049, 95% CI −10.303 to −0.014). There is a negative significant correlation between affective symptoms and perceived visibility of characteristics and subjective attractiveness.

Moreover, 14.9% of the variance of quality of life is explained by the perceived visibility and subjective attractiveness. The model has a medium quality of fit (Cohen, 1988). It has been found that subjective attractiveness significantly influences quality of life at significance level 0.05 (β = 0.352, *p* = 0.002, 95% CI 0.370–1.577). There is a positive significant correlation between quality of life and perceived visibility of characteristics and subjective attractiveness. Results of the multiple regression are shown in Tables 2, 3 that are attached.

**TABLE 2:** Multiple regression sexual self-esteem and body image.

	Sexual self-esteem				Body image			
Predictor	*b*	*SE b*	β	*p*	*b*	*SE b*	β	*p*
VIS	1.224	0.625	0.22	0.057	1.301	0.612	0.193	0.037
ATTR	0.502	0.26	0.217	0.054	1.532	0.253	0.551	<0.001
*R* ^2^	0.129 (*p* = 0.004)				0.418 (*p* < 0.001)			
	95% CI −0.015 to 1.02				95% CI 0.082–2.519			
	95% CI −0.020 to 2.469				95% CI 1.028–2.036			

**TABLE 3:** Multiple regression affective symptoms and quality of life.

	Affective symptoms				Quality of life			
Predictor	*b*	*SE b*	β	*p*	*b*	*SE b*	β	*p*
VIS	−5.159	2.584	−0.196	0.049	0.619	0.733	0.092	0.401
ATTR	−5.271	1.05	−0.493	<0.001	0.973	0.303	0.352	0.002
*R* ^2^	0.353 (*p* < 0.001)				0.156 (*p* < 0.001)			
	95% CI −10.303 to −0.014				95% CI −0.841 to 2.078			
	95% CI −7.361 to −3.182				95% CI 0.370–1.577			

## Discussion

The main focus in this study was to explore the relation between NF1 and sexuality in affected people. A relation between the visible symptoms of the disease and sexuality has been shown. NF1 affected people have a lower sexual self-esteem and a more negative body image compared to a healthy comparative group.

Furthermore, there was a moderate effect of disease visibility and subjective attractiveness on sexual self-esteem, body image, affective symptoms, and quality of life. A tendency showed that those affected living in a partnership have a more pronounced sexual self-esteem. This suggests that partnership might function as a protective factor. This tendency was already shown by Leidger et al. (2019).

Neurofibromatosis type 1 affected people are influenced in their sexuality by their disease. Due to the fact that the body image is considered to develop largely during puberty (Roth, 1998) and the symptoms of NF1 develop between childhood and puberty, an early disturbance of the development of the body image could be assumed. As a consequence of the fact that those affected by NF1 often have the impression of not meeting a common ideal of beauty, a low self- esteem can be the result.

In the present sample, persons affected by NF1 have more pronounced depressive symptoms than a healthy comparative group. This is in concordance with the study from Cohen et al. (2015). They have shown that a total of 55% of the NF1 patients investigated showed evidence of clinical depression (Cohen et al., 2015).

Our findings showed as well that NF1 affected people report a lower pronounced quality of life than a healthy control group. This is consistent with the study from Kodra et al. (2009). They examined the quality of life of 129 NF1 individuals and showed that the NF1 patients had a lower quality of life than a norm sample (Kodra et al., 2009).

Neurofibromatosis type 1-patients with major skin involvement have reduced skin related quality of life. Within them, current findings suggest experimentally, that the higher the use of dysfunctional coping, the more quality of life, psychological stress and self-esteem are affected (Bottesi et al., 2020). This study suggests that NF1 patients with high skin involvement and dysfunctional coping skills possibly benefit from psychological counseling with coping treatments aimed improving perceived self-efficacy and learning more adaptive coping strategies (Bottesi et al., 2020).

It should be noted that due to the anonymity of the questionnaire there was no possibility of a selection, no assurance of clinical accuracy (only self-report) and the degree of disease was determined with no extend of skin manifestation.

In addition, the high dropout rate of the participants was a disadvantage. The dropout rate among NF1 patients was 71.78 and 54.60% among the control group.

Possibly the high dropout rate of both groups is caused by the intimacy of the topic. Although an increased sexualization of the public context can be observed in advertising and media, personal sexuality is still largely taboo and is usually communicated less openly in private. Sexuality related questions can easily trigger shame and thus create a feeling of discomfort. However, the anonymous online questionnaire offered a protected space for the topic’s intimacy. The high drop-out rate of NF1 patients could also be partially caused by the increased occurrence of cognitive deficits caused by the disease (Soucy et al., 2012). These cognitive deficits could affect attention performance and concentration, which may have made it difficult to complete the 30-min questionnaire.

The results of the present study suggest that sexuality in NF1 patients has so far been insufficiently understood. There is clearly a need of sexuality-related counseling and interventions if indicated. A condition related to sexual health can lead to stress in the social environment of those affected. Given the fact that healthcare providers are not addressing intimacy concerns in the NF1 community (Cannon et al., 2019), it appears useful for health professionals to openly address the topic of sexuality in consultation hours and as such attempt to relieve patients of inhibitions and fears.

One possible method of support for NF1 patients with sexuality-related issues is the development of an NF1-specific questionnaire for the assessment of sexuality. This questionnaire can be used in medical consultation hours and included in NF1 treatment interventions. A psoriasis-specific part for the sexual medicine questionnaire for chronic diseases (SFCE) exists for psoriasis. The partners of the patients were also interviewed in the study to develop the questionnaire (Sharav, 2012). It is also useful for a further questionnaire to consult the partners of NF1 affected people to involve them in a possible therapy. A partnership could function as a productive factor.

This study provides new insights into the sexuality of people affected by NF1. It appears to be relevant considering the sexuality of NF1 persons as an integral part of a comprehensive health care approach.

## Data Availability Statement

The raw data supporting the conclusions of this article will be made available by the authors, without undue reservation.

## Ethics Statement

The studies involving human participants were reviewed and approved by the Ethikkommission der Ärztekammer Hamburg. The patients/participants provided their written informed consent to participate in this study.

## Author Contributions

AL, MV, V-FM, and TN conceived the presented idea. MV developed the theory, performed the computations, and wrote the manuscript with support from AL and V-FM. TN and V-FM helped to supervise the project. All authors discussed the results and contributed to the final manuscript.

## Conflict of Interest

The authors declare that the research was conducted in the absence of any commercial or financial relationships that could be construed as a potential conflict of interest.

## Publisher’s Note

All claims expressed in this article are solely those of the authors and do not necessarily represent those of their affiliated organizations, or those of the publisher, the editors and the reviewers. Any product that may be evaluated in this article, or claim that may be made by its manufacturer, is not guaranteed or endorsed by the publisher.

## Footnotes

1
www.bv-nf.de
